# Crisis leadership behaviors in healthcare: survey validation and influence on staff outcomes in primary care clinics during the COVID-19 pandemic

**DOI:** 10.1186/s12913-024-11061-5

**Published:** 2024-05-07

**Authors:** Michelle Yang, Jenna M. Evans, Sara J. Singer, Jennifer Gutberg, Tracy H. Porter, Agnes Grudniewicz

**Affiliations:** 1 École interdisciplinaire des sciences de la santé/Interdisciplinary School of Health Sciences, université d’Ottawa /University of Ottawa, 25 University Private, Ottawa, ON K1N 7K4 Canada; 2 DeGroote School of Business, McMaster University, Hamilton, ON L8S 4E8 Canada; 3 Department of Health Policy, Stanford University, 615 Crothers Way, Stanford, CA 94305 USA; 4 Institute of Health Policy, Management, and Evaluation, University of Toronto, 155 College St, Toronto, ON M5T 3M6 Canada; 5 Monte Ahuja College of Business, Cleveland State University, 1860 E. 18th St, Cleveland, OH 44114 USA; 6 Telfer School of Management, University of Ottawa, 55 Laurier Ave. E, Ottawa, ON K1N 6N5 Canada

**Keywords:** Pandemic, Health systems, Leadership, Virtual care, Telehealth, Psychometrics

## Abstract

**Background:**

The COVID-19 pandemic triggered an unprecedented transition from in-person to virtual delivery of primary health care services. Leaders were at the helm of the rapid changes required to make this happen, yet outcomes of leaders’ behaviours were largely unexplored. This study (1) develops and validates the Crisis Leadership and Staff Outcomes (CLSO) Survey and (2) investigates the leadership behaviours exhibited during the transition to virtual care and their influence on select staff outcomes in primary care.

**Methods:**

We tested the CLSO Survey amongst leaders and staff from four Community Health Centres in Ontario, Canada. The CLSO Survey measures a range of crisis leadership behaviors, such as showing empathy and promoting learning and psychological safety, as well as perceived staff outcomes in four areas: innovation, teamwork, feedback, and commitment to change. We conducted an exploratory factor analysis to investigate factor structure and construct validity. We report on the scale’s internal consistency through Cronbach’s alpha, and associations between leadership scales and staff outcomes through odds ratios.

**Results:**

There were 78 staff and 21 middle and senior leaders who completed the survey. A 4-factor model emerged, comprised of the leadership behaviors of (1) “task-oriented leadership” and (2) “person-oriented leadership”, and select staff outcomes of (3) “commitment to sustaining change” and (4) “performance self-evaluation”. Scales exhibited strong construct and internal validity. Task- and person-oriented leadership behaviours positively related to the two staff outcomes.

**Conclusion:**

The CLSO Survey is a reliable measure of leadership behaviours and select staff outcomes. Our results suggest that crisis leadership is multifaceted and both person-oriented and task-oriented leadership behaviours are critical during a crisis to improve perceived staff performance and commitment to change.

**Supplementary Information:**

The online version contains supplementary material available at 10.1186/s12913-024-11061-5.

## Background

 The COVID-19 pandemic renewed interest in crisis leadership in healthcare. Crisis leadership is “a process in which leaders act to prepare for the occurrence of unexpected crises, deal with the salient implications of crises, and grow from the disruptive experience of crises” [1]. With little warning, healthcare leaders had to rapidly develop, communicate, and execute an organizational response to the COVID-19 pandemic [2]. In primary care settings, leaders implemented remote work arrangements and switched from in-person to virtual care delivery to minimize virus exposure while maintaining effective patient care [2]. Within days, primary care clinics and other healthcare organizations were offering virtual care options to their clients – a digital transformation unlike any ever seen before in the healthcare industry [3]. The behaviours leaders exhibited (and continue to exhibit) in responding to the pandemic shaped individual and organizational outcomes, including the success and sustainability of innovative virtual programs [4]. COVID-19 incentivized policy makers and healthcare leaders to move from the slow “reimagining” of healthcare to rapid “recreation” of entire health systems through adoption of telemedicine [2]. Yet, many organizations have, or will, rebound to in-person service delivery after the pandemic, resulting in the loss of innovative and successful virtual programs [5]. How leaders perceive a crisis, communicate about it, and respond to it shapes staff perceptions [6] and influences how well the organization adapts to the crisis [7, 8]. This study (1) developed and validated the Crisis Leadership and Staff Outcomes (CLSO) Survey in primary care and (2) investigated the leadership behaviours exhibited during the transition to virtual care and their influence on perceived staff performance and staff commitment to sustaining organizational changes post-pandemic.

When leaders view a crisis as a dilemma or threat, they may inadvertently stifle staff motivation and learning [7, 8]. Conversely, when leaders view a crisis as an opportunity, they may promote staff experimentation and innovation [7, 8]. There is a lack of attention in the scholarly literature to the latter. Much research on leading during crises focuses on leader behaviour related to damage control, rather than leader behaviour related to positive adaptations [7]. After a crisis has subsided, an organization may rebound to pre-crisis conditions, reflecting ‘first order change’ and demonstrating single loop learning – a form of ‘quick-fix’ learning in which problems are corrected without questioning the underlying conditions that caused them [9]. Alternatively, an organization can exhibit double loop learning and ‘second order’ change that addresses the root causes of problems and transforms the fundamental properties of the organization to prevent future problems [8, 10]. While both forms of learning are important, double-loop learning is more likely to maximize long-term performance [9]. Leaders’ behaviours play a key role in whether single- or double-loop learning occurs. Recent studies emphasize that those leading during crisis recovery must be forward-thinking, incorporating innovative systems into the rebuilding process rather than merely seeking to “return to normal” [2, 11, 12].

Previous studies have examined how leadership behaviours influence a wide range of outcomes such as quality of care, staff well-being, and staff turnover during crises; some have even explored the relationship between those behaviours and the sustainability of organizational transformations [13–16]. In the current pandemic, studies have found that leaders influenced effective allocation and use of Personal Protective Equipment (PPE), staff redeployment, disaster management, and development of innovative approaches in operational strategy [11]. Yet, little attention has been paid to the impact of leadership behaviours on staff commitment to changes necessitated by the COVID-19 pandemic, such as the mandatory shift to virtual care within primary care settings which occurred worldwide [2]. In other words, it is unclear how leadership promotes or inhibits transformational organizational change in the context of a crisis such that ‘second order’ change can occur [11]. Moreover, some research on transformational change has predominantly taken an exploratory and interpretive approach [17], for example by using qualitative interviews to learn how leaders mitigate disasters [18]. Because there is less data validating findings statistically, there is a need for quantitative measurement tools to complement qualitative investigations to assess leadership during crises comprehensively and generate more generalizable findings.

Quantitative measures have been used during and following previous crises to assess leadership both within and outside the healthcare industry (Table 1). For example, the Conjoint Community Resiliency Assessment Measure (CCRAM) was used to measure leaders’ ability to assess, monitor, and enhance community resilience following crisis situations [19]. The Crisis Leader Efficacy in Assessing and Deciding scale (C-LEAD) was developed to capture the efficacy of leaders to assess information and make decisions in public health and safety crises [20]. A survey developed by Balasubramanian and Fernandes [10] measured the competencies of leaders and resulting employees’ satisfaction with their organizations’ effectiveness in handling the COVID-19 pandemic. Also during the COVID-19 pandemic, Al-Asfour [19] utilized the Crisis Leadership Survey (CLS) and Servant Leadership Questionnaire (SLQ) to find a predictive relationship between servant leadership and higher educational institutions officials’ level of readiness for the pandemic. These surveys, used both before and during the current pandemic context, have provided insight into the influence of leaders’ behaviours and decision-making practices during the various phases of crises. However, these surveys and their applications do not examine the influence of leaders’ behaviours on staff perceived performance or staff commitment to crisis-induced changes. Furthermore, many of these surveys rely on leader self-ratings, which introduce bias into measurement, and many were developed outside of healthcare and contain language that does not translate to the healthcare context [21]. For these reasons, the development and application of a new survey was required to answer our research questions.

**Table 1:** Survey Instruments on Crisis Leadership

Reference	Instrument Name	Description	Dimensions	Sample Items
Al-Asfour et al., 2022 [22]	Servant Leadership Questionnaire (SLQ); Crisis Leadership Survey (CLS)	SLQ measures frequency with which an individual believes they exhibit servant-leader qualities (rated by employees); CLS measures pandemic crisis readiness response (self-rated by leaders)	SLQ: Five factor solution • Altruistic calling • Emotional healing • Wisdom • Persuasive mapping • Organizational stewardship CLS: single factor structure	• This person believes that our organization needs to function as a community (SLQ) • I empower, equip and enable employees (CLS) • I establish a clear vision and direction (CLS)
Adamu and Mohamad, 2019 [23]	Internal Crisis Communication (ICC)	Measures employee perceptions of crisis communication from managers in their organization (rated by employees)	Single factor structure	• Management involves employee representatives in the crisis management team • Management tried to reduce employees’ anxiety during crisis
Balasubramanian and Fernandes, 2022 [24]	Crisis Leadership Model	Measures the competencies of leaders and resulting employees’ satisfaction with their organizations’ effectiveness in handling the COVID-19 pandemic (rated by employees)	Seven factor structure: • Compassion and Care • Openness and Communication • Adaptiveness • Resilience and Courage • Decisiveness • Consultation and Collaboration • Empowerment	• Have encouraged employees to openly and honestly express their concerns • Have tried new and unconventional means to overcome the crisis
Hadley et al., 2009 [20]	Crisis Leader Efficacy in Assessing and Deciding scale (C-LEAD)	Measures the efficacy of leaders in assessing information and making decisions in public health and safety crises (self-rated by leaders)	Single factor structure	• I can modify my regular work activities instantly to respond to an urgent need • I can make decisions and recommendations even when I don’t have as much information as I would like
Leykin et al., 2013 [25]	Conjoint Community Resiliency Assessment Measure (CCRAM)	Measures leaders’ ability to assess, monitor, and enhance community resilience following crisis situations (rated by community residents)	Relevant sub-scale ‘Leadership’ (single factor structure) examines perceptions of local leadership during times of crisis	• I have faith in the decision makers in the municipal authority • The municipal authority (regional council) provides its services in fairness
Bhanja et al., 2022 [26]	Leadership and burnout scale	Measures relationship between healthcare leadership attributes and clinician burnout during the COVID-19 pandemic (rated by employees)	Three dimensions: • Process clarity • Leader inclusiveness • Joint problem solving	• Hospital leadership has made appropriate efforts to respond to input from frontline workers during COVID • Leadership communicated about this crisis in a way that helped me do my job
Lim et al., 2020 [27]	Emergency preparedness and response readiness: *Leadership*	Measures impact of disaster management preparedness, leadership, and group integration on response readiness for an earthquake (rated by employees)	Single leadership construct	• I am confident that the leaders will take the lead in the frontline response during an emergency • I trust the leaders consider my safety in the emergency work during earthquake • I need leaders to direct our work during an emergency
Abenir et al., 2022 [28]	Toolkit for Measuring Community Disaster Resilience (MCDR): *Governance* thematic area	Measures factors that contribute to or impede the success of community-based leadership to achieve disaster resilience (rated by community members)	Six resilience components: • Existence, effectiveness, commitment, and accountability • Rights and awareness advocacy • Disaster Risk Reduction (DRR) Integration with development planning • Access to funding and partnerships • Inclusion of vulnerable groups • Women’s participation	• Is the community leadership committed, effective, and accountable? • Are the vulnerable groups in the community included/ represented in community decision making and management?
Obeng, 2022 [29]	Leadership Practices Inventory (LPI)	Measures school administrators’ self-reported leadership abilities and performance during a crisis (self-rated by leaders)	Five dimensions: • Model the way • Inspired a shared vision • Challenge the process • Enabling Others to Act • Encourage the heart	• I give people a great deal of freedom and choice in deciding how to do their work • I search outside the formal boundaries of my organization for innovative ways to improve what we do
Klebe et al., 2021 [30]	Health-Oriented Leadership (HoL) Scale: *Staff Care*	Measures leadership behaviors that improve or maintain staff health during crisis (rated by employees)	Three dimensions: • Health awareness • Value of health • Health behavior	• My direct supervisor notices when I need a break • My supervisor invites me to inform him/her about health risks at my workplace • It is important for my supervisor to reduce health risks at my workplace
Eichenauer et al., 2022 [31]	Perceived agency and communality	Measures frequency with which leader exhibited each behaviour when managing crisis situations (rated by employees)	Two dimensions: • Agentic • Communal	• [Leader] argues until co-workers see the ideas • [Leader] goes beyond self-interest for the good of the employees • [Leader] expresses concern with subordinates that are going through difficult times
Senbeto and on, 2021 [32]	Crisis Leader Efficacy (CLE)	Measures leaders’ intention and capability to examine information, readiness to change, and the surrounding changing trends (self-rated by leaders)	Single factor structure	• I can anticipate interpersonal ramifications of my decisions • I can summarize the key issues involved in a situation to others regardless of how much data I have

In this study we developed and validated a new survey instrument, the Crisis Leadership and Staff Outcomes (CLOS) Survey, in primary care clinics operating during the COVID-19 pandemic. We also used the results of the survey to examine the leadership behaviours exhibited during the transition to virtual care and their influence on perceived staff performance and staff commitment to sustaining organizational changes post-pandemic. We used staff perceptions of their leaders’ behaviours during the onset of the COVID-19 pandemic as the primary source of data for this study since previous research emphasizes that to gain a deep understanding of crisis leadership, we must consider staff’s first-hand experiences of being led during the crisis period [12, 33, 34]. Our survey can be used by researchers and practitioners to investigate leadership influence on staff perceived performance and staff commitment to crisis-induced change, allowing for inter-organizational and inter-jurisdictional comparisons. The results also provide healthcare leaders with actionable insights on leadership behaviours that support positive staff outcomes, particularly commitment to lasting structural change and innovation.

## Methods

The CLSO Survey was tested amongst a sample of leaders and staff working in select Community Health Centres in the province of Ontario, Canada. Community Health Centres (CHCs) are not-for-profit, community-governed primary healthcare organizations in Ontario [35]. Interprofessional teams at the 74 CHCs serve populations with lower socioeconomic status, offering clinical primary care services as well as an array of social services (i.e., domestic violence prevention and recovery support, parenting education, addictions counseling, nutrition and cooking classes) [35]. All methods were carried out in accordance with relevant guidelines and regulations, and approved by the University of Ottawa Research Ethics Board (Reference number: S-10-20-6202). Informed consent was obtained from all adult subjects prior to their participation. Written consent was obtained before data collection and survey administration.

### Scale development and properties

The CLSO Survey was originally developed as a 34-item questionnaire by a self-organized group of scholars formed in response to COVID-19 through the Academy of Management Health Care Division (Additional Files 1 and 2). A rapid review of leadership and crisis leadership surveys was conducted to identify relevant theories, concepts, and items. Survey topics were prioritized and items modified through multiple rounds of interviews and iteration with leaders from two health systems who volunteered to participate. Core concepts retained for measuring leadership behaviours included: promoting teamwork, communicating, offering feedback, empowering, promoting learning and psychological safety, and showing empathy (Additional File 3). Core concepts retained for measuring staff outcomes included: innovation outcomes, teamwork outcomes, feedback outcomes, and commitment to change (Additional File 3). Although the final items were inspired and informed by existing scales (e.g., [36, 37]), they underwent significant editing for clarity and relevance by the research and practitioner team such that only one portion of the final survey is based on a validated scale; the commitment to change outcome items in our survey are based on the change-related commitment questionnaire [38] with modifications to specify the change as the shift to virtual care delivery. All items were modified to specify the crisis context as the COVID-19 pandemic.

We administered two versions of the CLSO Survey, one for staff and one for leaders, and combined the data for survey validation. The two variations were deployed with minimal wording differences: one for leaders to self-rate their behaviours and perceived outcomes, and one for staff members to rate their perceptions of leadership behaviours and perceived outcomes (Additional Files 1 and 2). We defined “leaders” as being senior executives (i.e., Executive Director, CEO, VP) or middle managers (i.e., Director, Lead). “Staff” included administrative and support staff as well as frontline service providers (i.e., nurses, physicians). However, a low baseline number of leaders at each CHC contributed to a low sample size of completed leader surveys. Therefore, we only present findings from the exploratory analyses of the staff survey, but we validated findings against the leaders’ survey to establish construct validity.

Each item of the CLSO Survey was answered retrospectively in reference to experiences between March – May 2020. Participants were asked to reflect on this three-month period because it was the most acute period of drastic and rapid change following the World Health Organization’s declaration on March 11, 2020 that the COVID-19 outbreak was a global pandemic [39]. The survey used a 5-point Likert scale with questions 1–15 following frequency scale response categories [1 = never, 2 = once, 3 = a few times, 4 = many times, 5 = almost always] and questions 16–34 following agreement scale response categories [1 = strongly disagree, 2 = disagree, 3 = undecided, 4 = agree, 5 = strongly agree]. The survey was administered via Survey Monkey for a period of 3 months from March – June 2021. Due to purposeful negative wording, we reverse-scored 4 items before running statistical analysis: “How often did this leader make decisions before securing broad consensus or buy-in?”; “The potential benefits of this change are not worth the costs in time and resources required to sustain it.”; “It is unrealistic to expect that we will sustain this change”; “It wouldn’t take much for me to abandon this change”. Following reverse-coding, higher scores on the scale represented greater positive perceptions of leadership behaviours or staff outcomes of leadership behaviours during the virtual shift of the COVID-19 pandemic.

### Sampling

Our sample was comprised of leaders and staff from 4 CHCs. These four were chosen as they represented the sites with the 2 highest and 2 lowest aggregate scores on a short ‘pulse’ version of the CLSO Survey, which was comprised of 7 items and administered to all 74 CHCs in the province. The administration of the CLSO Survey at these sites was part of a broader mixed methods study investigating crisis leadership behaviours (not yet published). Studying CHCs at opposing ends of the leadership behaviour spectrum reflects a validated sampling technique that offers sharper contrasts and more valuable insights than a random sample [40]. Participants from the selected sites were recruited via purposive and snowball sampling [41]. Staff and leaders were eligible to participate if they were employed at the CHC at the time of data collection and had worked at the CHC during the first 3 months of the COVID-19 pandemic from March to May 2020 [39].

### Statistical analysis

We tested the psychometric properties of CLSO Survey on SPSS-28 software using descriptive statistics and measures of internal consistency, exploratory factor analysis (EFA), construct validity, and regression analysis. We only included cases with over 50% survey completion rate.

We described sample characteristics (gender, years at CHC, and role at CHC) in Tables 2 and 3. Internal consistency was assessed using Cronbach’s alpha, using a cut-off of *r* = .7 as a reference for high reliability [42]. In calculations for scale performance, means of Likert responses (mean out of 5) were used instead of sum of responses. We used this because the response categories with no value (i.e., “unsure”, “not available”) were left out of the analysis since including these could have resulted in a lower sum of responses – an inaccurate measure of overall ratings.

**Table 2:** Staff Summary Statistics and Multiple Regression of Background Predictors

	*n* (%)	Mean item score (SD)	*R* ^2^ (SE)
**Gender**			0.012 (0.61)
Female	72 (92.3%)	3.84 (0.62)	
Male	6 (7.7%)	4.09 (0.41)	
Other	0 (0%)	N/A	
**Years at CHC**			0.147 (0.58)
Less than 1 year	5 (6.4%)	4.24 (0.31)	
1–5 years	36 (46.2%)	3.81 (0.65)	
6–10 years	14 (17.9%)	3.89 (0.57)	
11–15 years	9 (11.6%)	3.37 (0.58)	
More than 15 years	14 (17.9%)	4.15 (0.44)	
**Role Type**			0.097 (0.57)*
Administration	15 (19.2%)	3.98 (0.42)	
Interprofessional team	13 (16.7%)	3.82 (0.76)	
Coordination and navigation	11 (14.1%)	4.08 (0.39)	
Nurse	26 (33.3%)	3.74 (0.58)	
Primary care provider	5 (6.4%)	3.75 (0.99)	
Community worker	8 (10.3%)	4.27 (0.32)	
*Total*	78	3.86 (0.61)	

*p < .05

*SD* Standard Deviation, *SE* Standard Error, *CHC* Community Health Center, *N/A* Not Available

**Table 3:** Leader Summary Statistics

	*n* (%)
**Gender**	
Female	17 (81%)
Male	4 (19%)
Other	0
**Years at CHC**	
1–5 years	6 (28.6%)
6–10 years	4 (19%)
11–15 years	6 (28.6%)
More than 15 years	5 (23.8%)
**Leadership level**	
Senior executive	6 (28.6%)
Middle manager	15 (71.4%)
*Total*	21

We employed EFA on the original 34 items to investigate the scale’s factor structure and construct validity in lieu of confirmatory factor techniques, which would require a larger sample size to be statistically robust [43]. We obtained factor loadings by principal components analysis (PCA) with varimax rotation and the Anderson-Rubin procedure, which produced factor scores that are uncorrelated and standardized, with a mean of zero and standard deviation of one [44]. We examined performance of the CLSO Survey with factor analysis using six criteria: (i) factors should have eigenvalues ≥ 1 [45], (ii) each item should load ≥ 0.4 on the primary factor [43], (iii) item loadings should have a difference of ≥ 0.2 between components [46], (iv) there should be ≥ 3 items loading on each factor [43], (v) R^2^ (communalities) should be ≥ 0.4 [47], (vi) the coefficient alpha of each factor should be ≥ 0.7 [46]. The factor solution is presented in Additional File 4.

We used correlation matrices and factor loadings from the EFA to assign items to factors. Then, we averaged the items to create a summary score for the factor (Additional File 4). The constructs of the factors were discussed amongst our research team; those pertaining to leadership styles (independent variables) were regressed onto staff outcomes (dependent variables) to find if there was a predictive relationship. Model fit, goodness-of-fit, and model effects were assessed using the likelihood ratio chi-square test and Pearson chi-square test [43, 48, 49] (Table 4). The odds ratio (OR) of higher outcome scores and respective 95% Confidence Interval (CI) associated with relevant leadership behaviours were calculated using generalized linear modeling (GLM). Alpha was set at 5% and all probability tests reported were two-tailed. Additionally, the independent variables were modeled simultaneously in a multiple regression to account for the relative contribution of multiple predictors in staff outcomes (Tables 5 and 6). We entered factors into the ordinary least squares regression models in stages, using hierarchical (blockwise) entry. Further, to assess if there were any main effects of background characteristics, we regressed categorical predictors (years at CHC, gender, staff role category) onto the results of the overall CLSO Survey, tabulating means across selection categories (Tables 2 and 3). Finally, we compared the staff survey’s factor solution and internal consistency to the results of the leader’s survey to investigate construct validity.

**Table 4:** Model Fit and Odds of Higher Outcome Scores

Independent variables		**Commitment to sustaining change**	**Performance self-evaluation**
**Task-oriented leadership**	Model fit *Likelihood chi-square (df)*	3.65 (1)*	13.14 (1)**
	Goodness-of-fit *Pearson chi-square*	891.59 (799)	437.77 (479)
	Odds Ratio (95%CI)	1.67 (0.99-2.82)*	2.62 (1.55-4.42)**
**Person-oriented leadership**	Model fit *Likelihood chi-square (df)*	4.47 (1)*	10.09 (1)**
	Goodness-of-fit *Pearson chi-square*	847.71 (819)	521.65 (503)
	Odds Ratio (95%CI)	1.66 (1.04-2.65)*	2.05 (1.31-3.22)**

*p < .05, **p < .01

*df* Degrees Freedom, *CI* Confidence Interval

**Table 5:** Regression Models Predicting Staff Outcome (Task-oriented leadership as the first predictor)

	Model 1				Model 2			
	Commitment to sustaining change		Performance self-evaluation		Commitment to sustaining change		Performance self-evaluation	
	B (SE)	ß	B (SE)	ß	B (SE)	ß	B (SE)	ß
**Task-oriented leadership**	0.26 (0.10)	0.28*	0.24 (0.07)	0.38***	0.15 (0.16)	0.16	0.18 (0.10)	0.29
**Person-oriented leadership**					0.13 (0.14)	0.16	0.07 (0.09)	0.12
F-ratio	6.360		12.802		3.649		6.646	
R^2^	0.077		0.146		0.089		0.152	
Adjusted R^2^	0.065		0.134		0.064*		0.129**	

*p < .05, **p < .01, and ***p < .001

*SE* Standard Error

**Table 6:** Regression Models Predicting Staff Outcome (Person-oriented leadership as the first predictor)

	Model 1				Model 2			
	Commitment to sustaining change		Performance self-evaluation		Commitment to sustaining change		Performance self-evaluation	
	B (SE)	ß	B (SE)	ß	B (SE)	ß	B (SE)	ß
**Person-oriented leadership**	0.23 (0.09)	0.28*	0.19 (0.06)	0.34	0.13 (0.14)	0.16	0.18 (0.10)	0.29
**Task-oriented leadership**					0.15 (0.16)	0.16	0.07 (0.09)	0.12
F-ratio	6.441		9.809		3.649		6.646	
R^2^	0.078		0.116		0.089		0.152	
Adjusted R^2^	0.066		0.104		0.064*		0.129**	

*p < .05, **p < .01

*SE* Standard Error

## Results

### Descriptive statistics

A summary of our sample is presented in Tables 2 (staff) and 3 (leaders). A total of 78 staff and 21 leaders comprised our sample. Most staff and leaders were female (92.3% and 81%, respectively). Most staff had worked at the CHC for 1–5 years (46.2%) and leaders were nearly equally distributed across years of experience (1–15 + years). We identified six employee role categories: administration (i.e., administrative assistant, medical receptionist, data management staff), interprofessional team (i.e., dietician, social worker, occupational therapist, health promoter), coordination and navigation (i.e., intake worker, telemedicine coordinator), nurse (i.e., registered practical nurse, registered nurse, nurse educator), primary care provider (i.e., family physician, nurse practitioner), and community worker (i.e., community health worker, community developer), with nurses representing the largest category (33.3%). There were 6 (28.6%) senior executives and 15 (71.4%) middle managers in our sample.

In this sample of staff from 4 CHCs (*n* = 78), 3.87 (SD = 0.61) out of 5 was the average item score. On average, male staff had higher scores than females. Staff who worked at their CHC for less than a year had the highest scores, followed by those who worked at their CHC for more than 15 years, 6–10 years, 1–5 years, and 11–15 years, in this order. Averages across staff role type categories showed that community workers had the highest scores, followed by those in coordination and navigation roles, administration, interprofessional team, primary care providers, and nurses, in this order. Out of the 3 background variables (gender, years at CHC, staff role type), only years at CHC was a significant predictor of performance on the scale, explaining 14.7% of the variance (*p* = .019).

### Internal consistency

Assessment of the internal consistency of the CLSO Survey demonstrated that the scale had a high reliability coefficient (Cronbach’s alpha = 0.930). Only a few items improved alpha if the item was deleted (highest = 9.350).

### Exploratory factor analysis

The Kaiser-Meyer-Olkin (KMO) measure of sampling adequacy (0.842) was above the acceptable cut-off of 0.5, supporting the use of exploratory factor analysis (EFA) for this sample. Bartlett’s test (χ²=916.682, df = 435, *p* < .001) was significant, indicating inter-item correlations are sufficiently strong for an EFA.

Using criteria (i) (eigenvalues ≥ 1), 7 factors emerged. However, a visual test of the slopes indicated a plateau following an inflection point at the fifth factor. This would justify the retention of 4 factors [43]. Further, the 7-factor model did not meet the criteria of at least 3 items loading ≥ 0.4 for each factor as the last three factors only had 1 valid item-loading each.

#### Retaining factors

A second analysis was run to extract only 4 factors. The resulting 4-factor model met criteria (i), (iv), and (vi). However, there were cross loadings on 4 items (“how often did this leader ask about work-related problems you were experiencing?”, “how often did this leader seek input from you about changes they were considering?”, “this leader sought input from me about what communication I felt was needed”, and “how often did this leader thank you for raising concerns”). Additionally, item 9 (“how often did this leader reveal they were not doing well emotionally”) did not meet any of the criterion, justifying its removal as a method of maintaining psychometric quality [43]. The next step was to run the analysis again, retaining 4 factors and excluding this item. Following this, the 4-factor model had minimal cross-loadings and exceeded all other criteria. To explore if there may have been more than 1 latent construct for the first factor, as it was larger than the rest, we applied an EFA to the items comprising this factor and examined its correlation matrix. The factor loadings and inter-item correlations were not significant, providing support for the validity of the 4-factor model. Additional File 4 summarizes the individual factor loadings.

#### Identifying constructs

We analyzed the questions within each factor, finding common themes among highly loading questions to identify what the constructs are. We identified two leadership behaviours (factors 1 and 2) and two outcomes (factors 3 and 4) (Additional File 4). The items comprising factor 1 were related to task-oriented leadership. These items measured teamwork, communication, and framing of the crisis. The items comprising factor 2 were related to person-oriented leadership, focusing on leaders’ encouragement, empathy, and empowerment of those they led during the crisis and subsequent organizational changes. We thus named these leadership factors “task-oriented leadership” and “person-oriented leadership”, respectively. The items comprising Factor 3 were related to staff commitment to change, including their perception of the underlying goals of the change, the costs versus benefits of the change, how likely it is the organization will sustain the change, and their personal willingness to commit to or abandon the change. The items comprising Factor 4 were related to their perceptions of their own performance, including how well they worked with others and improved processes and how innovative and responsive they were. We thus named these staff outcomes “commitment to sustaining change” and “performance self-evaluation”, respectively.

Upon identification of the factors and team discussions of item assignment, we removed 3 items for theoretical- or results-based reasons. As an example, question 7 (“how often did this leader ask about work-related problems you were experiencing?”) did not fit as well as the other items in factor 1 with the teamwork or communication constructs and significantly cross-loaded. Additionally, question 2 (“how often did this leader invite you to share suggestions or concerns?”) seemed redundant as its constructs were already captured by items in factor 2. Question 8 (“how often did this leader ask about your emotional well-being?”) significantly cross-loaded and was related to empathy; however, in a crisis context, it is unclear if higher or lower ratings on this item are related to better leadership outcomes. Additional File 5 presents the final list of 30 questions on the CLSO Survey following these adaptations.

### Regression analysis

#### Commitment to sustaining change models

The model with task-oriented leadership as a predictor for positive outcomes on commitment to sustaining change significantly improved fit relative to the null model [χ² (1) = 3.648, *p* = .046]. Goodness-of-fit Pearson chi-square tests were non-significant, showing the model was a good fit to our sample data [χ²(799) = 891.598, *p* = .12]. Increases on task-oriented leadership were significantly associated with increases in commitment to sustaining change (OR = 1.667, 95%CI = 0.986–2.818). With person-oriented leadership as the predictor, the model fit the data well [χ² (1) = 4.471, *p* = .034]. Goodness-of Fit Pearson chi-square test was non-significant, showing good model fit [χ²(819) = 847.710, *p* = .236]. Increases on person-oriented leadership were significantly associated with positive increases in commitment to sustaining change (OR = 1.658, 95%CI = 1.036, 2.654) (Table 4).

#### Performance self-evaluation models

The model with task-oriented leadership as a predictor for positive outcomes on performance self-evaluation significantly improved fit relative to the null model [χ² (1) = 13.140, *p* < .001]. Goodness-of-fit Pearson chi-square tests was non-significant, showing our model was a good fit to our sample data [χ²(479) = 437.765, *p* = .912]. Increases on task-oriented leadership were significantly associated with increases in performance self-evaluation (OR = 2.615, 95%CI = 1.549, 4.415). With person-oriented leadership as the predictor, the model fit the data well [χ² (1) = 10.093, *p* = .001]. Goodness-of Fit Pearson test was non-significant, showing good model fit [χ²(503) = 521.654, *p* = .274]. Increases on person-oriented leadership were significantly associated with positive increases in performance self-evaluation (OR = 2.054, 95%CI = 1.308,3.224) (Table 4).

#### Multivariable models

To find a linear combination of both predictors for the two leadership outcomes, we conducted hierarchical regression, entering factors into the regression models in two stages. In the first hierarchical regression (Table 5), we entered task-oriented leadership as the first predictor and person-oriented leadership as the second. In the second hierarchical regression (Table 6), we entered person-oriented leadership as the first predictor and task-oriented leadership as the second.

The final model with both predictors significantly explained 6.4% of the variation in scores on commitment to sustaining change, and 12.9% of the variance in performance self-evaluation (*p* = .031; *p* = .002, respectively). In Model 1 where either of the leadership styles is entered first hierarchically, both task-oriented and person-oriented leadership styles were significant predictors independently (*p* < .05). However, in both hierarchical regressions, when the second predictor was entered to form Model 2, the effects of either leadership style became nonsignificant (*p* > .05).

### Construct validity

We validated the factors that we found in the staff survey against the leader survey. The EFA suggests a 4-factor solution of the CLSO Survey. Amongst staff, this solution explained 62.65% of the variance in the scale, and comparably, amongst leaders, it explained 61.41% of the variance. The alphas of the scales amongst both groups were excellent and comparable (staff: 0.80–0.94; leaders: 0.61–0.93). Moreover, the alphas of the individual factors of both scales were considered strong [50].

## Discussion

Leadership style is an integral factor shaping staff and organizational responses to crises and subsequent commitment to change [13], but few validated measurement tools exist to support research and practice in this area. We developed the CLSO Survey to define and measure crisis leadership behaviours and their impact on staff’s perceived performance and their commitment to sustaining innovation in primary care in Canada. We tested the CLSO Survey during the first year of the COVID-19 pandemic, which triggered an unprecedented and transformative transition to virtual care delivery. Virtual care revolutionized access to healthcare services and was a solution to overcome barriers imposed by pandemic restrictions [51]. However, the success and sustainability of this novel healthcare modality is contingent on leadership behaviours and staff perceptions. In this study, we examined leadership as an influence on staff commitment to these changes during and potentially beyond this pandemic as well as staff perceived performance.

Testing the psychometric properties of the CLSO Survey addresses the need to conduct quantitative studies to validate themes and test conceptual models of crisis leadership [33]. In this study, we bridged this gap through developing a novel measure that can be used as a tool to evaluate leadership during a crisis and validating it to support its utility and encourage widespread use. Moreover, specifically focusing on leadership during the virtual care transition spurred by the pandemic highlights the issue of sustaining positive change following a crisis, rather than returning to the pre-crisis status quo.

We examined perceptions of leadership behaviours and their impact on perceived performance and commitment to change during the transition to virtual care delivery in four Ontario CHCs with a sample of 78 staff. Our analysis revealed that the CLSO Survey had strong psychometric properties. We found excellent reliability metrics of the full scale as it had a high alpha coefficient. Correlation analysis, factor analysis, and team consensus on the relevance of poorly-loaded items to measuring the theoretical construct indicated which items were redundant to measuring leadership in our context; this justified the removal of 4 items, rendering the full scale to be 30 final items (Additional File 5).

Our factor analysis supported the 4-dimensional nature of the survey. “Task-oriented leadership”, “person-oriented leadership”, “commitment to sustaining change”, and “performance self-evaluation” were found to be the factor solution that describes the leadership style and impact CHC leaders had on their staff during the transition to virtual care during the COVID-19 pandemic. There is a linear relationship between the two leadership behaviours and the two staff outcomes; higher scores on task-oriented leadership behaviour and person-oriented leadership behaviour each individually correlated with higher self-ratings on staff’s commitment to sustaining change, and performance self-evaluation. The subscales also had strong reliability coefficients comparable to the overall alpha. Additionally, when we tested the 4-factor solution among two populations (staff and leaders), we found evidence for the scale’s construct validity, showing it was a true measure of leadership constructs.

Many leadership studies have established a dichotomy between two overarching categories of leadership: (1) “task-focused” styles that facilitate accomplishment of tasks and (2) “person-focused” styles that facilitate the development and interaction of staff [52, 53]. The leadership behaviour constructs that emerged from our analysis mirror this dichotomy. In particular, items in our “task-oriented leadership” dimension (questions 1–10) related to the frequency and quality of feedback staff received from their leaders, and how the leaders provided praise, rewards, and transparent role expectations (Additional File 5). This is in line with Burn’s [54] theory that behaviours resembling task-oriented leadership usually focus on relationships based upon reward contingencies. This, in turn, was related to the outcome variables, staff commitment to sustaining virtual care and how they perceived their performance during this shift; these associations may occur because transactional or task-focused behaviours tend to be used by leaders in completing organizational requirements of managing human resources [53], which mirrors what was seen during the shift to virtual primary care delivery.

Another value of task-oriented leadership is leaders who exemplify this style make decisions that provide a sense of control [55]. This leadership quality was critical during the virtual shift of COVID-19 as providers were pressured to quickly adopt digital tools to safely and effectively provide patient care [54, 56]. It is possible that leaders who exhibited task-oriented leadership behaviours were more successful at building confidence, thereby enhancing commitment to the new modalities of work [55]. Findings relating to this dimension of the leadership scale suggest that effective leadership fosters clear communication and team building. This could be done through reviewing and clarifying staff roles and responsibilities and supporting staff in functioning in teams. The scale items relating to communication on the task situation also suggest framing is a key leadership strategy. How a leader frames the crisis situation affects staff’s understanding of both the situation and its significance within the organization. The results of our study suggest framing a disaster as an improvement opportunity rather than a hindrance may increase staff commitment to the implementation and sustainment of virtual services, despite the time and resources required.

The items that comprised our second “person-oriented leadership” dimension (questions 11–18) relate to how leaders involved staff in change processes (Additional File 5). This supports the literature on how transformational leadership is linked to staff motivational states, two-way communication, and empowerment [53, 57]. Consequently, these behaviours facilitate effective team performance to solve a complex problem. This may explain how person-oriented leaders in our study were more likely to motivate sustainability of virtual care during the pandemic; according to Burke [50], the degree of motivation translates to how well staff can operate within environments that require adaptive behaviour, which is related to innovation and learning [58].

Moreover, studies have found that person-oriented leaders motivate their staff by recognizing and respecting their needs [59], which reduces staff strain and burnout [16]. This may explain why person-oriented leadership was particularly effective during the COVID-19 pandemic as staff had to work under stressful and demanding circumstances as they navigated a new virtual care delivery system [2, 51]. Through the use of our survey, we found that person-oriented leaders were more likely to honor the efforts and perspectives of their staff through listening to their concerns or suggestions, using them to guide changes and decisions, and finally, reporting back on what happened to their suggestions or thanking staff for making suggestions. In turn, we saw increases in staff’s self-rated performance, enhancing self-efficacy in their roles and potentially improving work processes in ways that will have lasting effects beyond the current crisis. This finding also suggests that effective crisis leadership is not solely a hierarchical process, but instead, leaders have the opportunity to empower others to be leaders in their own way by shaping the change process.

The uncertainty of a widespread disaster exacerbates the challenges associated with leadership [55, 60]. Previously, Geier [58] found that task-focused leadership was the dominant predictor of staff’s performance in emergencies. Leaders were perceived to display less person-focused leadership during extreme events compared to everyday events as this facilitated active monitoring of actions and application of new courses of action when the crisis necessitated it [60]. In contrast, other empirical studies suggest that person-focused leadership behaviours are particularly effective for service teams in the context of change, in part because person-oriented leadership facilitates learning [58]. These discrepancies in the literature indicate each leadership style has strengths and shortcomings when managing a crisis. Person-oriented leadership may not be appropriate in periods of time constraint due to the time it takes to build consensus [12]. Conversely, a task-driven leader may lack empathy and interpersonal skills when managing a crisis that affects the health and safety of organizational members [12]. Therefore, there is no one-size-fits-all approach during crises; based on our analysis and supporting literature, we recommend leaders take an all-encompassing approach rather than be overly oriented towards one specific leadership style. The regression models built in this study further support this sentiment. The constructs measured by the CLSO Survey show us that crisis leadership is multifaceted, and both person-oriented and task-oriented leadership are critical in a disaster setting, as higher scores on either of these behaviours equally and significantly predicted positive outcomes. Therefore, our analysis suggests an effective leader leading during COVID-19 should be able to display multiple competencies and styles as a coherent package. This entails that leaders remain adaptable and alert to when certain leadership orientations may be warranted- individually or combined- during the crisis. During a pandemic, such as COVID-19 that has come in waves and necessitated organizational changes in different phases, this ability is important as different sets of responses have and will be needed to manage and plan the different stages of a crisis [61]. In this way, crisis leadership can be viewed in a context-specific way in order to be truly effective.

### Limitations and future directions

There are several limitations to this study. First, only four organizations participated in the survey. However, these CHCs were purposefully selected to maximize variation in leadership behaviours based on a ‘pulse’ version of our survey. Second, while our sample size (*n* = 78) yielded an excellent KMO statistic, indicating an EFA was appropriate, it did not have enough statistical power to conduct a confirmatory factor analysis (CFA) [43]. Future work should include a sample size appropriate for a CFA in order to confirm the 4-factor solution found in this study, screen for any alternative models, and lend further evidence to construct validity. Third, we only interpreted the results of the staff survey in this study due to sampling limitations with the middle and senior leaders who participated. The perspectives of leaders and how they self-describe their leadership behaviours and self-rate their performance leading during crises are also vital to informing a model on how crisis leadership affects perceived team performance and the sustainability of changes post-disaster. Future research should incorporate the views of not only middle and senior leaders, but also front-line clinical leaders who are closest to the point of care and whose behaviours during a crisis could vary from leaders at other hierarchical levels and may help further explain staff outcomes. Fourth, the survey was administered 12 months after the inception of the COVID-19 pandemic. As such, our data may be skewed by recall bias. Due to the nature of the pandemic and its impact on healthcare organizations, it was not possible for us to collect data earlier. To avoid this in the future, we recommend administering this survey closer to the emergence of the crisis event. Fifth, it is unknown if the constructs captured by the CLSO Survey are applicable to other disasters or crises necessitating different types of organizational adaptations (other than a shift to virtual care). There is more work needed to validate the generalizability and transferability of the 4-factor model to different crises with different populations. Sixth, each question on the survey had 5 response categories that were scored, with the addition of one or two unscored categories (i.e., Unsure, Don’t remember, Not Applicable). Therefore, although the overall score of the scale should be 150, much of the analysis relied on the average item score (out of 5) as a measure of performance since unscored responses could inaccurately lower one’s overall score. This limits the establishment of valid cut-off scores and is generally a less rigorous way of assessing psychometric data; an average item score may not be an accurate reflection of overall experiences nor perceptions, especially if the scale is used in the context of comparing the performance of two or more groups. A future direction may be to establish cut-offs for organizational and research purposes. Cut-off scores can be used to differentiate between groups (i.e., low performers, high performers) or can guide interventions. Seventh, the use of a cross-sectional dataset limits our ability to infer causality between effects of the independent variables related to leadership styles on the dependent variables related to staff outcomes. Eighth, we followed the opinions of some scholars that a Kaiser-Meyer-Olkin analysis for sampling adequacy is sufficient to support the use of an EFA for a particular sample size [43] (Field, 2013). However, we recognize that other scholars have suggested at least 10 participants per variable [62], or over 300 participants [63], both of which we did not achieve. For that reason, future work using a larger sample size may be important for confirming the findings of our study. Finally, future work should include qualitative methodology for phenomenological insight into the validity of leadership constructs explored in our survey. This is important as both quantitative and qualitative findings are needed to build comprehensive understanding of leadership behaviours and outcomes.

In addition to being used for research purposes, the CLSO Survey could be used by organizations as a training or assessment tool before, during, and after a crisis to explore leaders’ behaviours and assess their impact on the success of organizational changes during a crisis. Such assessment may also be a pre-emptive measure to improve leadership before a crisis emerges. To do this, practitioners need only to replace references to the COVID-19 pandemic in the survey with another crisis.

## Conclusion

In this exploratory study, we found that a new scale, the CLSO Survey, was reliable in measuring leadership behaviours and select staff outcomes during a novel crisis, the COVID-19 pandemic, and is a valid tool to describe the leadership behaviours that influence staff commitment to crisis-induced transformative changes in primary care settings. The results of our analysis also lend further support to the notion that transformational, person-oriented and transactional, task-oriented leadership styles are *both* strong levers for effectively managing and sustaining changes in organizations during crises [34, 64]. The results suggest that both leadership styles must be integrated to form a comprehensive and effective organizational response. Overall, our results expand knowledge on leadership behaviour constructs and their specific outcomes during crises and associated organizational changes.

### Supplementary Information

**Additional file 1:** Original CLOS Survey – Staff Version.

**Additional file 2:** Original CLOS Survey – Leader Version

**Additional file 3:** CLOS Survey – Intended Underlying Theories and Concepts

**Additional file 4:** CLOS Survey – Factor Loadings

**Additional file 5:** Final CLOS Survey

## Data Availability

The data are not publicly available but are available from the corresponding author on reasonable request.

## Abbreviations

PPE: Personal Protective Equipment
SLQ: Servant Leadership Questionnaire
CLS: Crisis Leadership Survey
CLSO: Crisis Leadership and Staff Outcomes Survey
ICC: Internal Crisis Communication
C-LEAD: Crisis Leader Efficacy in Assessing and Deciding scale
CCRAM: Conjoint Community Resiliency Assessment Measure
MCDR: Measuring Community Disaster Resilience
LPI: Leadership Practices Inventory
HoL: Health-Oriented Leadership
CLE: Crisis Leader Efficacy
CHC: Community Health Centre
EFA: Exploratory Factor Analysis
OR: Odds Ratio
CI: Confidence Interval
GLM: Generalized Linear Modeling
SD: Standard Deviation
KMO: Kaiser-Meyer-Olkin statistic
CFA: Confirmatory Factor Analysis
