# Supplementary material for: Crisis leadership behaviors in healthcare: survey validation and influence on staff outcomes in primary care clinics during the COVID-19 pandemic
Source: BMC Health Serv Res. 2024 May 7;24:590. doi: 10.1186/s12913-024-11061-5 (PMC11075262; doi:10.1186/s12913-024-11061-5)
Supplement: Supplementary file 1 — Additional file 1: Original CLOS Survey – Staff Version. [file 12913_2024_11061_MOESM1_ESM.docx]

# **Crisis Leadership and Staff Outcome Survey – Staff Version**

**Instructions**

Thank you for participating in the COVID-19 Leadership Survey – Staff Version. Please only complete this version of the survey if you are a **provider or staff member in a** **non-leadership role**. The purpose of the survey is to examine the behaviours leaders exhibited in managing the organizational crisis spurred by the pandemic, particularly the **rapid transition to virtual delivery of services**.

The survey asks about your work and experience **during approximately March to May 2020, when COVID-19 was a new, grave concern in your area**. Please refer to this period when answering the questions. The survey will take approximately **10-15 minutes** to complete.

We encourage your candid responses. Your answers are confidential and will not be identifiable in any resulting publication. Your answers will only be reported in aggregate, with answers combined into groups large enough to protect against individual identification.

Your decision to complete and return this survey is an indication of your consent to participate. If you wish to withdraw for any reason, you may do so by closing the survey. After you have submitted the survey, it will no longer be possible to withdraw your data from the study as it has not been linked with any identifying information.

At the end of the survey, you may enter your contact information if you wish to be entered into a draw for a $100 gift certificate.

**Follow-Up Interviews**

We will be conducting interviews with CHC staff who have provided virtual **social** services. At the end of the survey, you will have an opportunity to provide your contact information if you are interested in participating in an interview. Whether you participate or not will not be shared with anyone in your organization.

# Your answers will not be identifiable and will only be analyzed in aggregate.

# **Please enter the name of your CHC:**

# ___________________________

**Please enter your role/position in the CHC:**

# ___________________________

**How many years have you been working with this CHC?**

-Less than 1 year

-1-5 years

-6-10 years

-11-15 years

-More than 15 years

**What is your gender?**

-Female

-Male

-Other (Please specify): ________

In the questions below, we use the term ‘leaders’ to refer to both senior executives and middle managers at your CHC. Please think about the **leader you see or interact with most** and choose the response that best describes their behaviour.

1. How often did this leader express that COVID-19 presents a unique opportunity to improve the way the CHC does things?

*Never, Once, A Few Times, Many Times, Almost Always, Unsure/Don’t Remember*

1. how often did this leader invite you to share suggestions or concerns?

*Never, Once, A Few Times, Many Times, Almost Always, Unsure/Don’t Remember*

1. how often did this leader thank you for raising concerns?

*Never, Once, A Few Times, Many Times, Almost Always, Unsure/Don’t Remember, N/A*

1. how often did this leader act on your suggestions?

*Never, Once, A Few Times, Many Times, Almost Always, Unsure/Don’t Remember, N/A*

1. how often did this leader report back on what happened with your suggestions?

*Never, Once, A Few Times, Many Times, Almost Always, Unsure/Don’t Remember, N/A*

1. how often, when addressing you, did this leader explicitly frame the context as a safe space for disagreement?

*Never, Once, A Few Times, Many Times, Almost Always, Unsure/Don’t Remember*

1. how often did this leader ask about work-related problems you are experiencing?

*Never, Once, A Few Times, Many Times, Almost Always, Unsure/Don’t Remember*

1. how often did this leader ask about your emotional well-being?

*Never, Once, A Few Times, Many Times, Almost Always, Unsure/Don’t Remember*

1. how often did this leader reveal they were not doing well emotionally?

*Never, Once, A Few Times, Many Times, Almost Always, Unsure/Don’t Remember*

1. how often did this leader provide feedback to guide your work?

*Never, Once, A Few Times, Many Times, Almost Always, Unsure/Don’t Remember*

1. how often did this leader provide feedback to guide your team’s work?

*Never, Once, A Few Times, Many Times, Almost Always, Unsure/Don’t Remember*

1. how often did this leader communicate with you about changes being implemented?

*Never, Once, A Few Times, Many Times, Almost Always, Unsure/Don’t Remember*

1. how often did this leader explain *why* changes were being made, not just *what* changes were being made?

*Never, Once, A Few Times, Many Times, Almost Always, Unsure/Don’t Remember*

1. how often did this leader seek input from you about changes they were considering?

*Never, Once, A Few Times, Many Times, Almost Always, Unsure/Don’t Remember*

1. how often did this leader make decisions before securing broad consensus or buy-in?

*Never, Once, A Few Times, Many Times, Almost Always, Unsure/Don’t Remember*

1. this leader encouraged me to make changes I felt were important.

*Strongly Disagree, Disagree, Undecided, Agree, Strongly Agree, Don’t Remember*

1. this leader established a regular frequency of communication with me.

*Strongly Disagree, Disagree, Undecided, Agree, Strongly Agree, Don’t Remember*

1. this leader sought input from me about what communication I felt was needed.

*Strongly Disagree, Disagree, Undecided, Agree, Strongly Agree, Don’t Remember*

1. this leader reviewed roles and responsibilities with me.

*Strongly Disagree, Disagree, Undecided, Agree, Strongly Agree, Don’t Remember*

1. this leader took action, as new individuals were assigned to their groups, to make sure we functioned as a “real team”.

*Strongly Disagree, Disagree, Undecided, Agree, Strongly Agree, Don’t Remember, N/A*

1. this leader called attention to the strengths of each person on our team.

*Strongly Disagree, Disagree, Undecided, Agree, Strongly Agree, Don’t Remember*

1. this leader ensured that we agreed on ways we work together as a team.

*Strongly Disagree, Disagree, Undecided, Agree, Strongly Agree, Don’t Remember*

1. I was committed to the implementation of virtual services.

*Strongly Disagree, Disagree, Undecided, Agree, Strongly Agree, Don’t Remember*

1. I found new ways to innovate any time I was faced with a constraint.

*Strongly Disagree, Disagree, Undecided, Agree, Strongly Agree, Don’t Remember*

1. I improved work processes in ways that will have lasting effects beyond this crisis.

*Strongly Disagree, Disagree, Undecided, Agree, Strongly Agree, Don’t Remember*

1. I worked effectively with my team.

*Strongly Disagree, Disagree, Undecided, Agree, Strongly Agree, Don’t Remember*

1. I worked effectively with other teams across the CHC.

*Strongly Disagree, Disagree, Undecided, Agree, Strongly Agree, Don’t Remember*

1. I was very responsive to feedback.

*Strongly Disagree, Disagree, Undecided, Agree, Strongly Agree, Don’t Remember*

**In the statements below the “change” refers to your CHC’s transition to virtual delivery of services due to COVID-19.**

1. The principles of this change effort are good goals to continue to shoot for.

*Strongly Disagree, Disagree, Undecided, Agree, Strongly Agree*

1. I am strongly committed to sustaining this change effort.

*Strongly Disagree, Disagree, Undecided, Agree, Strongly Agree*

1. The potential benefits of this change are not worth the costs in time and resources required to sustain it.

*Strongly Disagree, Disagree, Undecided, Agree, Strongly Agree*

1. It is unrealistic to expect that we will sustain this change.

*Strongly Disagree, Disagree, Undecided, Agree, Strongly Agree*

1. It wouldn’t take much for me to abandon this change.

*Strongly Disagree, Disagree, Undecided, Agree, Strongly Agree*

1. I am convinced we need to sustain this change at my CHC.

*Strongly Disagree, Disagree, Undecided, Agree, Strongly Agree*

This question is specific to the delivery of **social** services. Please describe a moment or action that best reflects your CHC’s leadership during the transition to virtual delivery of **social** services between March-May 2020, when COVID-19 was a new, grave concern in your area.

____________________________________________________________________________________________________________________________________________________________

As part of this study, we will be conducting virtual interviews with CHC leaders and staff involved in providing or managing virtual **social** services. If you would like to be considered for an interview, please provide your name and email address. Whether you participate or not will not be shared with anyone in your organization. Your personal information will not be linked to your survey responses.

Name: ______________ Email: ______________

To thank you for completing the survey, you are conducting a draw for a $100 gift certificate. To participate in this draw, please enter your name and email address below. Your personal information will not be linked to your survey responses.

Name: ______________ Email: ______________
