# Supplementary material for: Crisis leadership behaviors in healthcare: survey validation and influence on staff outcomes in primary care clinics during the COVID-19 pandemic
Source: BMC Health Serv Res. 2024 May 7;24:590. doi: 10.1186/s12913-024-11061-5 (PMC11075262; doi:10.1186/s12913-024-11061-5)
Supplement: Supplementary file 2 — Additional file 2: Original CLOS Survey – Leader Version [file 12913_2024_11061_MOESM2_ESM.docx]

# **Crisis Leadership and Staff Outcome Survey – Leader Version**

**Instructions**

Thank you for participating in the COVID-19 Leadership Survey: Long-Form. Please only complete this version of the survey if you are in a **leadership role**. The purpose of the survey is to examine the behaviours leaders exhibited in managing the organizational crisis spurred by the pandemic, particularly the rapid transition to **virtual delivery of services**.

The survey asks about your work and experience **during approximately March to May 2020, when COVID-19 was a new, grave concern in your area**. Please refer to this period when answering the questions. The survey will take approximately **10 minutes** to complete.

We encourage your candid responses. Your answers are confidential and will not be identifiable in any resulting publication. Your answers will only be reported in aggregate, with answers combined into groups large enough to protect against individual identification.

Your decision to complete and return this survey is an indication of your consent to participate. If you wish to withdraw for any reason, you may do so by closing the survey. After you have submitted the survey, it will no longer be possible to withdraw your data from the study as it has not been linked with any identifying information.

At the end of the survey, you may enter your contact information if you wish to be entered into a draw for a $100 gift certificate.

**Follow-Up Interviews**

We will be conducting interviews with CHC leaders and staff involved in providing or managing virtual **social** services. At the end of the survey, you will have an opportunity to provide your contact information if you are interested in participating in an interview. Whether you participate or not will not be shared with anyone in your organization.

# **Please enter the name of your CHC** (your answers will not be identifiable and will only be analyzed in aggregate)**:**

# ___________________________

**What is your role in the CHC?**

- Senior executive (e.g., Executive Director, CEO, VP)

- Middle manager (e.g., Director, Lead)

**How many years have you been working with this CHC?**

-Less than 1 year

-1-5 years

-6-10 years

-11-15 years

-More than 15 years

**What is your gender?**

-Female

-Male

-Other (Please specify): ________

**When COVID-19 was a new, grave concern in your area (March to May 2020) and your CHC had to transition to virtual delivery of services…**

1. roughly what percent of your time were you able to work on things you felt were truly important (rather than just urgent, requiring immediate attention)?

*Less than 25% of the time, 25% to less than 50% of the time, 50% to less than 75% of the time, more than 75% of the time*

1. roughly what percent of your time were you putting out fires rather than working on the CHC’s strategic priorities?

*Less than 25% of the time, 25% to less than 50% of the time, 50% to less than 75% of the time, more than 75% of the time*

1. roughly what percent of the time, when those you lead had an idea, were you able to provide resources (e.g., time, money)?

*Less than 25% of the time, 25% to less than 50% of the time, 50% to less than 75% of the time, more than 75% of the time*

1. how often did you express to those you lead that COVID-19 presents a unique opportunity to improve the way the CHC does things?

*Never, Once, A Few Times, Many Times, Almost Always, Unsure/Don’t Remember*

1. how often did you invite those you lead to speak up with a suggestion or concern?

*Never, Once, A Few Times, Many Times, Almost Always, Unsure/Don’t Remember*

1. how often did you thank those you lead for raising concerns?

*Never, Once, A Few Times, Many Times, Almost Always, Unsure/Don’t Remember*

1. how often did you act on suggestions you received from those you lead?

*Never, Once, A Few Times, Many Times, Almost Always, Unsure/Don’t Remember*

1. how often did you report back to those you lead about what happened with their suggestions?

*Never, Once, A Few Times, Many Times, Almost Always, Unsure/Don’t Remember*

1. when you addressed those you lead, how often did you explicitly frame the context as a safe space for disagreement?

*Never, Once, A Few Times, Many Times, Almost Always, Unsure/Don’t Remember*

1. how often did you ask those you lead about work-related problems they are experiencing?

*Never, Once, A Few Times, Many Times, Almost Always, Unsure/Don’t Remember*

1. how often did you ask those you lead about their emotional wellbeing?

*Never, Once, A Few Times, Many Times, Almost Always, Unsure/Don’t Remember*

1. how often did you let the those you lead know when you were not doing well emotionally?

*Never, Once, A Few Times, Many Times, Almost Always, Unsure/Don’t Remember*

1. how often did you make a decision without as much information as you would have liked?

*Never, Once, A Few Times, Many Times, Almost Always, Unsure/Don’t Remember*

1. how often did you do what you felt needed to be done, before securing broad consensus or buy-in?

*Never, Once, A Few Times, Many Times, Almost Always, Unsure/Don’t Remember*

1. how often did you reach out to someone who went through an earlier surge, pandemic, or crisis for advice about what you could do?

*Never, Once, A Few Times, Many Times, Almost Always, Unsure/Don’t Remember*

1. how often did you communicate with those you lead about changes being implemented?

*Never, Once, A Few Times, Many Times, Almost Always, Unsure/Don’t Remember*

1. how often did you explain *why* changes were being made, not just *what* changes were being made?

*Never, Once, A Few Times, Many Times, Almost Always, Unsure/Don’t Remember*

1. how often did you seek input from those you lead about changes you were considering?

*Never, Once, A Few Times, Many Times, Almost Always, Unsure/Don’t Remember*

1. how often did you provide those you lead with feedback to guide their work as a team?

*Never, Once, A Few Times, Many Times, Almost Always, Unsure/Don’t Remember*

1. how often did you provide those you lead with individual feedback to guide their work?

*Never, Once, A Few Times, Many Times, Almost Always, Unsure/Don’t Remember*

1. I encouraged those I lead to make changes they felt were important.

*Strongly Disagree, Disagree, Undecided, Agree, Strongly Agree, Don’t Remember*

1. I established a regular frequency of communication with those I lead.

*Strongly Disagree, Disagree, Undecided, Agree, Strongly Agree, Don’t Remember*

1. I sought input from those I lead about what communication they felt was needed.

*Strongly Disagree, Disagree, Undecided, Agree, Strongly Agree, Don’t Remember*

1. I reviewed roles and responsibilities with those I lead.

*Strongly Disagree, Disagree, Undecided, Agree, Strongly Agree, Don’t Remember*

1. I called attention to the strengths of each person on my team.

*Strongly Disagree, Disagree, Undecided, Agree, Strongly Agree, Don’t Remember*

1. I ensured that those I lead agreed on ways we work together as a team.

*Strongly Disagree, Disagree, Undecided, Agree, Strongly Agree, Don’t Remember*

1. those I lead were committed to the implementation of virtual services.

*Strongly Disagree, Disagree, Undecided, Agree, Strongly Agree, Don’t Remember*

1. those I lead found new ways to innovate any time they were faced with a constraint.

*Strongly Disagree, Disagree, Undecided, Agree, Strongly Agree, Don’t Remember*

1. those I lead improved work processes in ways that will have lasting effects beyond this crisis.

*Strongly Disagree, Disagree, Undecided, Agree, Strongly Agree, Don’t Remember*

1. those I lead worked together effectively.

*Strongly Disagree, Disagree, Undecided, Agree, Strongly Agree, Don’t Remember*

1. those I lead worked effectively with other teams across the CHC.

*Strongly Disagree, Disagree, Undecided, Agree, Strongly Agree, Don’t Remember*

1. those I lead were very responsive to feedback.

*Strongly Disagree, Disagree, Undecided, Agree, Strongly Agree, Don’t Remember*

**In the statements below the “change” refers to your CHC’s transition to virtual delivery of services due to COVID-19.**

1. The principles of this change effort are good goals to continue to shoot for.

*Strongly Disagree, Disagree, Undecided, Agree, Strongly Agree*

1. I am strongly committed to sustaining this change effort.

*Strongly Disagree, Disagree, Undecided, Agree, Strongly Agree*

1. The potential benefits of this change are not worth the costs in time and resources required to sustain it.

*Strongly Disagree, Disagree, Undecided, Agree, Strongly Agree*

1. It is unrealistic to expect that we will sustain this change.

*Strongly Disagree, Disagree, Undecided, Agree, Strongly Agree*

1. It wouldn’t take much for me to abandon this change.

*Strongly Disagree, Disagree, Undecided, Agree, Strongly Agree*

1. I am convinced we need to sustain this change at my CHC.

*Strongly Disagree, Disagree, Undecided, Agree, Strongly Agree*

This question is specific to the delivery of **social** services. Please describe a moment or action that best reflects your leadership during the transition to virtual delivery of **social** services between March-May 2020, when COVID-19 was a new, grave concern in your area. ____________________________________________________

As part of this study, we will be conducting virtual interviews with CHC leaders and staff involved in providing or managing virtual **social** services. If you would like to be considered for an interview, please provide your name and email address. Whether you participate or not will not be shared with anyone in your organization. Your personal information will not be linked to your survey responses.

Name: ______________ Email: ______________

To thank you for completing the survey, you are conducting a draw for a $100 gift certificate. To participate in this draw, please enter your name and email address below. Your personal information will not be linked to your survey responses.

Name: ______________ Email: ______________
