# Supplementary material for: Crisis leadership behaviors in healthcare: survey validation and influence on staff outcomes in primary care clinics during the COVID-19 pandemic
Source: BMC Health Serv Res. 2024 May 7;24:590. doi: 10.1186/s12913-024-11061-5 (PMC11075262; doi:10.1186/s12913-024-11061-5)
Supplement: Supplementary file 3 — Additional file 3: CLOS Survey – Intended Underlying Theories and Concepts [file 12913_2024_11061_MOESM3_ESM.docx]

***Crisis Leadership and Staff Outcomes Survey:***

Intended Underlying Theories and Concepts

**CRISIS LEADERSHIP BEHAVIOURS**

**Promoting teamwork**

- This leader took action, as new individuals were assigned to their groups, to make sure we functioned as a “real team”.
- This leader reviewed roles and responsibilities with me.
- This leader called attention to the strengths of each person on our team.
- This leader ensured that we agreed on ways we work together as a team.

**Communicating**

- This leader established a regular frequency of communication with me.
- This leader sought input from me about what communication I felt was needed.
- How often did this leader communicate with you about changes being implemented?
- How often did this leader seek input from you about changes they were considering?
- How often did this leader report back on what happened with your suggestions?
- How often did this leader explain *why* changes were being made, not just *what* changes were being made?

**Offering feedback**

- How often did this leader provide feedback to guide your work?
- How often did this leader provide feedback to guide your team’s work?

**Empowering**

- How often did this leader act on your suggestions?
- This leader encouraged me to make changes I felt were important.
- How often did this leader make decisions before securing broad consensus or buy-in?

**Promoting learning and psychological safety**

- How often, when addressing you, did this leader explicitly frame the context as a safe space for disagreement?
- How often did this leader invite you to share suggestions or concerns?
- How often did this leader thank you for raising concerns?
- How often did this leader express that COVID-19 presents a unique opportunity to improve the way the CHC does things?

**Showing empathy**

- How often did this leader ask about your emotional well-being?
- How often did this leader reveal they were not doing well emotionally?
- How often did this leader ask about work-related problems you are experiencing?

**STAFF OUTCOME 1 – Perceived performance**

***Innovation outcomes***

- I found new ways to innovate any time I was faced with a constraint.
- I improved work processes in ways that will have lasting effects beyond this crisis.

***Teamwork outcomes***

- I worked effectively with my team.
- I worked effectively with other teams across the CHC.

***Feedback outcomes***

- I was very responsive to feedback.

**STAFF OUTCOME 2 – Commitment to change**

- I was committed to the implementation of virtual services.
- The principles of this change effort are good goals to continue to shoot for.
- I am strongly committed to sustaining this change effort.
- The potential benefits of this change are not worth the costs in time and resources required to sustain it.
- It is unrealistic to expect that we will sustain this change.
- It wouldn’t take much for me to abandon this change.
- I am convinced we need to sustain this change at my CHC.
