# Supplementary material for: Crisis leadership behaviors in healthcare: survey validation and influence on staff outcomes in primary care clinics during the COVID-19 pandemic
Source: BMC Health Serv Res. 2024 May 7;24:590. doi: 10.1186/s12913-024-11061-5 (PMC11075262; doi:10.1186/s12913-024-11061-5)
Supplement: Supplementary file 4 — Additional file 4: CLOS Survey – Factor Loadings [file 12913_2024_11061_MOESM4_ESM.docx]

*Rotated Factor Analysis of the Crisis Leadership and Staff Outcome Survey*

|  | **Leadership behavior** | | | **Staff outcome** | |
| --- | --- | --- | --- | --- | --- |
|  | **Factor 1**  Task-oriented leadership | **Factor 2**  Person-oriented leadership | **Factor 3**  Commitment to sustaining change | | **Factor 4**  Performance self-evaluation |
| How often did this leader provide feedback to guide your team’s work? | **0.839** | 0.090 | | -0.046 | -0.012 |
| How often did this leader communicate with you about changes being implemented? | **0.833** | 0.096 | | 0.056 | -0.024 |
| How often did this leader provide feedback to guide your work? | **0.773** | 0.217 | | 0.115 | 0.051 |
| How often did this leader explain why changes were being made, not just what changes were being made? | **0.768** | 0.262 | | -0.087 | 0.117 |
| How often did this leader invite you to share suggestions or concerns? | **0.704** | 0.311 | | 0.227 | 0.080 |
| This leader established a regular frequency of communication with me. | **0.693** | 0.344 | | 0.186 | 0.145 |
| This leader took action, as new individuals were assigned to their groups, to make sure we functioned as a “real team”. | **0.686** | 0.315 | | 0.199 | 0.312 |
| This leader called attention to the strengths of each person on our team. | **0.656** | 0.445 | | 0.045 | 0.174 |
| This leader reviewed roles and responsibilities with me. | **0.630** | 0.288 | | 0.274 | 0.159 |
| This leader ensured that we agreed on ways we work together as a team. | **0.628** | 0.297 | | 0.202 | 0.282 |
| How often did this leader express that COVID-19 presents a unique opportunity to improve the way the CHC does things? | **0.597** | 0.112 | | -0.013 | 0.244 |
| How often did this leader ask about work-related problems you were experiencing? | **0.559** | 0.515 | | 0.132 | 0.056 |
| How often did this leader report back on what happened with your suggestions? | 0.188 | **0.803** | | -0.092 | 0.136 |
| This leader encouraged me to make changes I felt were important. | 0.288 | **0.695** | | 0.268 | 0.157 |
| How often did this leader act on your suggestions? | 0.225 | **0.694** | | 0.077 | 0.128 |
| This leader sought input from me about what communication I felt was needed. | 0.517 | **0.642** | | 0.219 | 0.134 |
| How often did this leader ask about your emotional well-being? | 0.433 | **0.598** | | 0.204 | 0.194 |
| How often, when addressing you, did this leader explicitly frame the context as a safe space for disagreement? | 0.235 | **0.596** | | -0.063 | -0.005 |
| How often did this leader thank you for raising concerns? | 0.475 | **0.593** | | -0.023 | 0.253 |
| How often did this leader seek input from you about changes they were considering? | 0.493 | **0.507** | | 0.252 | 0.303 |
| How often did this leader make decisions before securing broad consensus or buy-in? (Reverse scored) | 0.073 | **0.501** | | 0.123 | -0.207 |
| I am convinced we need to sustain this change at my CHC. | 0.103 | 0.109 | | **0.876** | 0.073 |
| It is unrealistic to expect that we will sustain this change. (Reverse scored) | 0.082 | 0.087 | | **0.847** | 0.018 |
| I am strongly committed to sustaining this change effort. | 0.229 | 0.122 | | **0.817** | 0.120 |
| It wouldn’t take much for me to abandon this change. (Reverse scored) | -0.017 | -0.005 | | **0.793** | 0.156 |
| The principles of this change effort are good goals to continue to shoot for. | 0.220 | 0.158 | | **0.736** | 0.036 |
| I was committed to the implementation of virtual services. | 0.196 | -0.062 | | **0.659** | 0.468 |
| The potential benefits of this change are not worth the costs in time and resources required to sustain it. (Reverse scored) | -0.075 | 0.036 | | **0.612** | 0.042 |
| I worked effectively with my team. | 0.097 | 0.039 | | 0.068 | **0.760** |
| I worked effectively with other teams across the CHC. | 0.071 | 0.074 | | 0.004 | **0.757** |
| I found new ways to innovate any time I was faced with a constraint. | 0.204 | -0.060 | | 0.196 | **0.743** |
| I was very responsive to feedback. | 0.111 | 0.222 | | 0.058 | **0.705** |
| I improved work processes in ways that will have lasting effects beyond this crisis. | 0.171 | 0.140 | | 0.292 | **0.591** |
