# Supplementary material for: Crisis leadership behaviors in healthcare: survey validation and influence on staff outcomes in primary care clinics during the COVID-19 pandemic
Source: BMC Health Serv Res. 2024 May 7;24:590. doi: 10.1186/s12913-024-11061-5 (PMC11075262; doi:10.1186/s12913-024-11061-5)
Supplement: Supplementary file 5 — Additional file 5: Final CLOS Survey [file 12913_2024_11061_MOESM5_ESM.docx]

*Crisis Leadership and Staff Outcome Survey (30 items)*

| **Sub-scales** | **Response Categories** |
| --- | --- |
| **Leadership behaviors**  *Please think about the leader you see or interact with most and choose the response that best describes their behavior.* | |
| ***Task-oriented leadership - 10 items*** | |
| This leader reviewed roles and responsibilities with me. | *Strongly Disagree, Disagree, Undecided, Agree, Strongly Agree, Don’t Remember* |
| This leader ensured that we agreed on ways we work together as a team. | *Strongly Disagree, Disagree, Undecided, Agree, Strongly Agree, Don’t Remember, N/A** |
| This leader called attention to the strengths of each person on our team. | *Strongly Disagree, Disagree, Undecided, Agree, Strongly Agree, Don’t Remember, N/A** |
| This leader took action, as new individuals were assigned to their groups, to make sure we functioned as a “real team”. | *Strongly Disagree, Disagree, Undecided, Agree, Strongly Agree, Don’t Remember, N/A** |
| How often did this leader provide feedback to guide your team’s work? | *Never, Once, A Few Times, Many Times, Almost Always, Unsure/Don’t Remember, N/A** |
| This leader established a regular frequency of communication with me. | *Strongly Disagree, Disagree, Undecided, Agree, Strongly Agree, Don’t Remember* |
| How often did this leader express that COVID-19 presents a unique opportunity to improve the way the CHC does things? | *Never, Once, A Few Times, Many Times, Almost Always, Unsure/Don’t Remember* |
| How often did this leader communicate with you about changes being implemented? | *Never, Once, A Few Times, Many Times, Almost Always, Unsure/Don’t Remember* |
| How often did this leader explain why changes were being made, not just what changes were being made? | *Never, Once, A Few Times, Many Times, Almost Always, Unsure/Don’t Remember* |
| How often did this leader provide feedback to guide your work? | *Never, Once, A Few Times, Many Times, Almost Always, Unsure/Don’t Remember* |
| ***Person-Oriented Leadership – 8 items*** | |
| How often did this leader seek input from you about changes they were considering? | *Never, Once, A Few Times, Many Times, Almost Always, Unsure/Don’t Remember* |
| How often did this leader act on your suggestions? | *Never, Once, A Few Times, Many Times, Almost Always, Unsure/Don’t Remember, N/A*** |
| How often did this leader report back on what happened with your suggestions? | *Never, Once, A Few Times, Many Times, Almost Always, Unsure/Don’t Remember, N/A*** |
| How often did this leader thank you for raising concerns? | *Never, Once, A Few Times, Many Times, Almost Always, Unsure/Don’t Remember, N/A*** |
| How often did this leader make decisions before securing broad consensus or buy-in? | *Never, Once, A Few Times, Many Times, Almost Always, Unsure/Don’t Remember* |
| This leader sought input from me about what communication I felt was needed. | *Strongly Disagree, Disagree, Undecided, Agree, Strongly Agree, Don’t Remember* |
| This leader encouraged me to make changes I felt were important. | *Strongly Disagree, Disagree, Undecided, Agree, Strongly Agree, Don’t Remember* |
| How often, when addressing you, did this leader explicitly frame the context as a safe space for disagreement? | *Never, Once, A Few Times, Many Times, Almost Always, Unsure/Don’t Remember* |
| **Staff outcome**  *In the statements below, the “change” refers to your CHC’s transition to virtual delivery of services due to COVID-19.* | |
| ***Commitment to Sustaining Change - 7 items*** | |
| I am convinced we need to sustain this change at my CHC. | *Strongly Disagree, Disagree, Undecided, Agree, Strongly Agree* |
| It is unrealistic to expect that we will sustain this change. | *Strongly Disagree, Disagree, Undecided, Agree, Strongly Agree* |
| I am strongly committed to sustaining this change effort. | *Strongly Disagree, Disagree, Undecided, Agree, Strongly Agree* |
| It wouldn’t take much for me to abandon this change. | *Strongly Disagree, Disagree, Undecided, Agree, Strongly Agree* |
| The principles of this change effort are good goals to continue to shoot for. | *Strongly Disagree, Disagree, Undecided, Agree, Strongly Agree* |
| I was committed to the implementation of virtual services. | *Strongly Disagree, Disagree, Undecided, Agree, Strongly Agree, Don’t Remember* |
| The potential benefits of this change are not worth the costs in time and resources required to sustain it. | *Strongly Disagree, Disagree, Undecided, Agree, Strongly Agree* |
| ***Performance Self-Evaluation -5 items*** | |
| I worked effectively with my team. | *Strongly Disagree, Disagree, Undecided, Agree, Strongly Agree, Don’t Remember* |
| I worked effectively with other teams across the CHC. | *Strongly Disagree, Disagree, Undecided, Agree, Strongly Agree, Don’t Remember* |
| I found new ways to innovate any time I was faced with a constraint. | *Strongly Disagree, Disagree, Undecided, Agree, Strongly Agree, Don’t Remember* |
| I was very responsive to feedback. | *Strongly Disagree, Disagree, Undecided, Agree, Strongly Agree, Don’t Remember* |
| I improved work processes in ways that will have lasting effects beyond this crisis. | *Strongly Disagree, Disagree, Undecided, Agree, Strongly Agree, Don’t Remember* |

*N/A denotes Not Applicable as employee did not work in a team

**N/A denotes Not Applicable as employee did not raise concerns/make suggestions
